# Social Network Types and Health among Older Adults in Rural China: The Mediating Role of Social Support

**DOI:** 10.3390/ijerph16030410

**Published:** 2019-01-31

**Authors:** Liping Ye, Xinping Zhang

**Affiliations:** School of Medicine and Health Management, Tongji Medical College, Huazhong University of Science and Technology, Wuhan 430030, China; xiaoye3531@126.com

**Keywords:** social network types, depressive symptoms, self-rated health, social support, rural older adults, cross-sectional study

## Abstract

This study aimed to identify social network types among older adults in rural China, to explore the relationship between social network types and the health of the older adults, and further, to examine the mediating role of social support in this relationship. A cross-sectional survey method was employed to investigate the health of adults aged 60 or older in rural areas of Hubei Province from 5 September 2018 to 15 October 2018; 405 samples were obtained. First, using *k*-means clustering analysis, we found five robust network types: diverse, restricted, family, friends and a specific type—family-restricted. Second, the results of multiple linear regression analysis showed that social network types were significantly associated with depressive symptoms and self-rated health in older adults. Older people with diverse friend networks were significantly associated with lower levels of depressive symptoms, whereas those with restricted and family-restricted networks were significantly associated with higher levels of depressive symptoms and lower levels of self-rated health. Finally, the results of multiple linear regression analysis confirmed that social support partially mediated the association of the identified social network types with depressive symptoms and self-rated health. Enriching the social network relationships of older adults and providing them with more social support should be conducive to promoting their mental and physical health.

## 1. Introduction

Social network relationships are considered to be an important factor affecting the health of older adults, and this view has been confirmed by many scholars [1,2,3]. Older adults can obtain friendship, support, relief, etc. by interacting with social network members, which is conducive to coping with stress and alleviating loneliness, thus promoting mental and physical health [3]. In particular, older adults who have lost the ability to take care of themselves, temporarily or for life, have a high degree of spiritual and material dependence on their social network members, and they require even more social support from these members [4]. Therefore, social network relationships are of central importance to older adults.

The social network type, which is a composite characterization of an individual’s social network relationships [5], consists of two components: the network structure (e.g., network size and composition) and interactions with network members (e.g., frequency of contact). The method of researching social network types was first proposed and used by Wenger; it initially identified five social network types in England and Wales, and served as diagnostic criteria for social work practice in older adults [5,6]. Litwin then effectively improved this method through a series of studies [5,7,8,9]. Social network typology is an emerging research field. Over time, the measurement indicators of social network types and the relationship between social network types and health have attracted the attention of many scholars. However, the research in this area is mostly concentrated in Western countries, such as the United States [10,11,12], Germany [13], Australia [14], and Israel [9,15]. The leading Asian country in this research area is South Korea [16,17,18,19], but there have been relatively few studies in China [20,21,22]. Cheng et al. [20] identified social network types among older Chinese adults living in Hong Kong and the networks’ relations to subjective well-being. Li et al. [21] used the national microdata to explore reciprocal associations between social network types and health. Additionally, Li et al. [22] analyzed the patterns and survival benefits of social network types. In these Chinese studies, the measurement indicators of social network types included both structural variables (e.g., social network size and frequency of interaction) and functional variables (e.g., instrumental support and emotional support) in deriving network types. However, more previous studies were just to adopt structural variables for clustering [4,9,10,11,12,14,15]. Therefore, the measurement indicators of social network types need to be further explored.

Among the identified social network types, the relatively consistent ones are ‘diverse’, ‘friends’, ‘family’, and ‘restricted’, which are found in many studies [10,12,13,14,17,20,21]. A ‘diverse’ network is characterized by a broad range of supportive relations with family, friends, and neighbours as well as frequent participation in social activities. A ‘friends’ network is characterized by frequent interactions with friends or neighbours but few kinship ties. In contrast, a ‘family’ network is characterized by frequent contact with family members, but little contact with friends and social activities. Finally, individuals with a ‘restricted’ network have limited social ties and low social activities. Some studies have developed subtypes based on the above four network types, such as ‘nonfamily’, ‘nonfriends’ [10], ‘diverse/family’, and ‘diverse/friends’ [18]. Some studies have identified unique social network types, such as a ‘distant family’ network characterized by few immediate kin but mostly distant kin in a sample of Hong Kong Chinese older adults [20], a ‘congregant’ network with frequent attendance at religious services among American older adults [23], and a ‘widowed’ network of women without a life partner among Mexican older adults [4]. There are certain differences in the social network types in different countries, mainly based on the unique social interpersonal milieu and cultural environment.

Previous studies have shown that social network types are significantly associated with health. Older adults in a diverse network are generally healthier than those in other social network types, whereas, those in a restricted network have worse health [10,14,16,17,19]. There are different opinions on the associations of friends and family networks with health. One view is that older adults with a friends network is better for the health than a family network. For example, in a study by Litwin [24], respondents in a friends network reported better morale than those in a family network. Park et al. [18] found the self-rated health and depressive symptoms were more favorable in a friends network than in a family network. Additionally, the three health indicators of loneliness, anxiety, and happiness were also more favorable in a friends network in a study by Litwin and Shiovitz-Ezra [5]. Another view is that a family network is better than a friends network. For example, Cheng et al. [20] found the subjective well-being of older adults in a family network to be better than that in a friends network. The different conclusions mentioned above may be related to different social and cultural contexts. For example, Fiori et al. [25] analyzed the relationship between social network types and the physical and mental health of older adults in the United States and Japan. Interestingly, while social networks types were related to health in the United States, no health differences according to network type were found in Japan.

Other scholars have analyzed the impact of social network types on health via additional pathways, such as mediating effects [10,26]. Webster et al. [26] examined the mediating role of social support in the association of social network types with depressive symptoms and health limitations, and found that social support only mediated the link between social network types and health limitations. In addition, the majority of studies analyzed the relationship between social networks and social support as well as the impact of social support on health [4,26,27,28]. These studies have suggested that social network types are closely related to social support, and older adults in a larger social network could receive more social support [4,28]. The research on whether social support mediates the link between social network types and health is need of further examination in diverse social contexts.

In summary, the current view of the empirical relationship between social network types and health has not yet reached a consensus, and the mechanism of how social network types affect health is still unclear. Although a large number of studies have confirmed that social network types are closely related to health, when compared with the studies of Western societies, the empirical research on the relationship between social network types and health of rural older adults in China is far from rich.

Chinese demographics signal that the country is gradually becoming an aging society. According to UN statistics, China’s population over 60 will reach 454 million by 2050 and account for 33% of the total, when it will be among those countries with the highest percentage of aging population in the world [29]. The geographic distribution of older adults in China is quite dispersed. Nearly 60% of older adults live in China’s economically disadvantaged rural areas, and the aging of rural populations is more advanced than urban ones [30]. The aging of the population will bring about various problems, such as depression and physical illness, which, in turn, will place great burdens on families and society [31]. The aging problem is ushering in great social changes, such as the shrinking of family size and the increase of rural-to-urban migration, which are also having significant impacts on the social network relationships of rural older adults [32]. Based on the unique socioeconomic circumstances of rural China, exploring the relationship between social network types and the health of rural older adults has important practical significance for improving the social network relationships of older adults and promoting their health.

In order to analyze the relationship between social network types and health, this paper is primarily founded on the following three questions: First, what are the primary social network types for rural older adults? Second, are different social network types associated with the health of older adults? Third, does social support play a mediating role in the relationship between social network types and health? This study mainly has two contributions: (1) Previous studies were mainly based on national microdata [18,21,22,33]. In contrast, this study designed the questionnaires and adopted a cross-sectional study approach to obtain the latest data on social network relationships and the health of rural older adults. Therefore, the survey content is more targeted and comprehensive. For example, some previous studies using microdata analysis have some limitations when describing the frequency of contact with social members only through face-to-face contact [18,33]. However, this study adds various ways of social contact (e.g., phone calls and video chats). (2) As yet, there is still no consensus on the empirical relationship between social network types and health, and how social network types impact health remains unclear. There is also a relative lack of research on the relationship between social network types and health among rural older adults in China. This study can make up for this deficiency and contribute to existing empirical evidence.

## 2. Data Sample and Methods

### 2.1. Data Sample

A cross-sectional survey method was conducted in six rural villages in Jiayu County, Hubei Province, China, from 5 September to 15 October 2018. Cluster sampling was conducted, the first being a random selection of villages, and six villages were selected by the lottery method from a total number of 14 villages in Paizhouwan town, Jiayu Country. The second sample was from a random selection of village groups, and one village group was selected from each village (average 10 groups in a village). Then we used a door-to-door survey to investigate all older adults of each village group, with the following inclusion criteria. (1) Local residents; (2) age ≥ 60 years old, referring to the criteria for defining older adults in Chinese authorities, such as China National Bureau of Statistics [34] and the China National Committee on Aging [35], and previous studies [10,16,20,26]; (3) normal intelligence, no problem with language communication; and (4) willing to participate in the survey. The questionnaire designed specifically for this study primarily includes items related to personal sociodemographic characteristics, social network, social support, depression, and self-rated health. After the face-to-face questionnaire survey, it was confirmed that each questionnaire was answered in full. Nearly 70 older adults in each village group were interviewed; a final sample of 405 respondents providing complete information was obtained. This study was approved by the Ethics Committee of Tongji Medical College, Huazhong University of Science and Technology (IORG: IORG 0003571).

### 2.2. Measures

#### 2.2.1. Sociodemographics

Firstly, the age of older adults was dichotomized to differentiate between younger (60–69 years old) and older individuals (≥70 years old). Gender was dichotomously coded (1 = male, 2 = female), as was marital status (1 = married, 2 = not married). Respondents who lived with their spouses or partners were considered married, and those who were widowed, divorced or separated, never married, or never lived with a partner were categorized as ‘not married’. Due to the low level of education in rural older adults, the educational levels were categorized as 1 = no education, 2 = primary school, and 3 = middle school or beyond. Household income was divided into four categories (unit: RMB): 1 = less than 5000, 2 = 5000–15,000, 3 = 15,000–25,000, and 4 = not less than 25,000.

#### 2.2.2. Social Network Variables

This study analyzed the mediating effects of social support on the relationship between social network types and health; therefore, this paper only included structural variables for clustering. Furthermore, considering that marital status would become a key factor in differentiating between network types and more distal relationship types that cannot be captured [20,33], we decided not to include marital status in social network type clustering. Seven social network variables were selected based on other network studies [10,23,33]: the number of living children, the number of close relatives, the number of close friends, the frequency of contact with living children, the frequency of contact with close relatives, the frequency of contact with close friends, and the frequency of social activities. The number of living children was obtained through a simple count (0, 1, 2, 3, 4, or ≥5). The number of close relatives and the number of close friends were summarized using a six-point scale: 0 (none), 1 (one), 2 (two to three), 3 (four to nine), 4 (10–20), or 5 (more than 20). This was designed to render the responses more comparable, as the number of close relatives or close friends were varied greatly [19,33]. The frequency of contact with living children, the frequency of contact with close relatives, and the frequency of contact with close friends were measured on a five-point scale ranging from 1 (more than once a year) to 5 (at least once a day), for instance, the number of living children was measured by the question *How often do you contact with living children through face-to-face, phone calls, video chats, etc.?* The frequency of social activities was measured by the question: *How often do you participate in social activities such as playing cards, playing mah-jong, square dancing, worshipping Buddha, going to church, etc.?* Responses were given on a five-point scale ranging from 1(never) to 5 (at least once a day). 

#### 2.2.3. Health Indicators

This study evaluated the health of older adults, including level of depressive symptoms and self-rated health. First, the depressive symptoms assessment was based on the 15-item Geriatric Depression Scale-Short Form (GDS-SF), developed by Sheikh and Yesavage [36], in the standard yes/no response format. The GDS-SF has a range of 0–15. Although the traditional cut-off for probable depression is 5 and higher [37], we used depression scores to evaluate depressive symptoms, with a higher score indicating more depressive symptoms (Cronbach’s α = 0.879). Second, a self-reported health indicator was selected from the relevant literature [21]. It was measured with a single question asking how respondents would rate their health using a five-point scale (1 = very poor; 5 = excellent).

#### 2.2.4. Social Support Variables

The 19-item Medical Outcomes Study Social Support Survey (MOS-SSS), developed by Sherbourne and Stewart [38], was used to measure social support. The scale assesses four dimensions of support: affective support, information and emotional support, positive social interaction, and tangible support. Responses to each item were recorded on a five-point scale (1 = never; 5 = always). In this study, the score for social support was the average score for all items, with a range of 1–5. The higher the score, the more social support was obtained (Cronbach’s α = 0.941).

### 2.3. Analytic Strategy

In order to answer the first research question, *k*-means cluster analysis was used to identify the social network type. The method first assigns an initial clustering center to each criterion variable and then iteratively updates until a predetermined number of optimal groupings have been achieved based on the distance between these clustering centers [39]. According to the principle of minimum Euclidean distance, the distance between clusters is measured, and it is confirmed that the homogeneity of the samples within each cluster is the highest and the sample heterogeneity between different clusters is the largest.

Furthermore, bivariate association analysis and multiple linear regression analysis were used to explore the second and third research questions. First, to examine whether the social network types differed regarding sociodemographics, depressive symptoms and self-rated health, we generated a series of univariate statistics. The differences of age, gender, married status, education level, and household income were analyzed using a chi-squared test, while the differences of depressive symptoms and self-rated health were subjected to analyses of variance (ANOVAs), together with Bonferroni post hoc tests. Then, the bivariate association between sociodemographics and health was tested via Spearman correlation analysis. Second, we examined the association of social network types with depressive symptoms and self-rated health. A stepwise multiple linear regression analysis with depressive symptoms and self-rated health as the dependent variables and social network types as independent variables was performed. In this step of analysis, the social network type was set as a dummy variable, and the diverse social network was used as a reference group. In order to control for any possible unequal distributions within clusters, the sociodemographic variables for age, gender, marital status, educational level, and household income were included as predictors. Finally, in order to explore whether social support played a mediating role in the relationship between social network types and health, this paper used the criteria proposed by Baron and Kenny for verification [40]. Considering that the mediation model had only one mediator and it was constructed mostly with observed variables, we chose the multiple regression analysis for mediation analysis. Additionally, this traditional mediation analysis has been used in previous relevant studies [10,26]. The statistical analysis in this study was based on SPSS software version 18.0 (IBM Corporation, Armonk, NY, USA).

## 3. Results

### 3.1. Social Network Clustering Analysis

In the findings of previous studies, four or more social network types were identified. In the cluster analysis, *k* was set to 4, 5, 6, and 7, respectively. In order to obtain ideal social network clusters, this study followed two clustering principles: (1) the number of cases in each cluster is reasonable and (2) the formed clusters should be meaningful. When *k* was set to 5, five robust network types were identified: diverse, restricted, family, friend, and a specific type—family-restricted. The distribution and characteristics of the social network types are shown in Table 1.

#### 3.1.1. Diverse Network Type

The value of each criterion variable of the diverse network was found to be relatively high: most close relatives, most frequent contact with close relatives and living children, and highest frequency of social activities.

#### 3.1.2. Restricted Network Type

The values of all criterion variables for the restricted network were the lowest. This type had the lowest proportion.

#### 3.1.3. Family-Restricted Network Type

The family-restricted network was a subtype of the restricted network, and its most prominent feature was that older adults in this type are close to their children. Among older adults in this type of network, the number of living children was the highest, and the frequency of contact with children was also high. However, older adults in this network type have less interaction with other social network members and rarely participate in social activities. 

#### 3.1.4. Family Network Type

The family network was characterized by frequent interactions with living children, and the number of living children was also high. However, the value of participation in social activities was low in this type, although it was better than in the family-restricted type. The family network represented the largest proportion.

#### 3.1.5. Friends Network Type

The most distinctive feature of the friends network was that older adults were in contact with close friends frequently. Older people in this type had the largest number of close friends and the most frequent contact with friends. At the same time, the value of participation in social activities was only exceeded by the diverse network.

### 3.2. Analysis of the Association between Social Network Type and Health

The results of the bivariate association between social network types and sociodemographics, depressive symptoms, and self-rated health are shown in Table 2. The sociodemographics of older adults in different social network types were quite different. The chi-squared test showed that age, gender, marital status, educational level, and household income were significantly different among different social network types (*p* < 0.05 or *p* < 0.001). For the variable of age, the older people (≥70 years old) were mostly distributed in the family-restricted network; the fewest were found in the friends network. Women accounted for 54.3% of the respondents. Moreover, women accounted for the highest proportion in the family-restricted network. The respondents were mainly married (80%), and the proportion of married individuals was highest in the diverse network, followed by the friends network, and lowest in the restricted network. Older adults in the diverse network had achieved the highest level of education. In contrast, older adults in the family-restricted network and restricted network had a relatively low level of education. In terms of household income, the diverse network and friends network had relatively high household incomes, while the restricted network had the lowest incomes. 

The results of ANOVAs showed that depressive symptoms and self-rated health were significantly different by social network types (*p* < 0.001). Bonferroni post hoc tests showed that older adults with diverse and friends networks were associated with a lower level of depressive symptoms than those with other networks, while the opposite was true for those older adults with restricted and family-restricted networks. Additionally, older adults with restricted and family-restricted networks were associated with lower levels of self-rated health than those with other networks, as expected.

The results of the bivariate relationship between sociodemographics and health are shown in Table 3, which indicate that depressive symptoms and self-rated health in older adults were closely related to sociodemographics. Being older, female or unmarried were positively correlated with depressive symptoms and negatively correlated with self-rated health. In addition, education level and household income were significantly negatively correlated with depressive symptoms and positively correlated with self-rated health.

The results of the multiple linear regression between social network types and health are shown in Table 4. In terms of depressive symptoms, older adults in restricted, family-restricted, and family networks displayed significantly higher levels of depressive symptoms than those in the diverse network (*p* < 0.001), but no significant difference was found between the friends network and the diverse network. In addition, marital status and household income were also two important factors affecting depressive symptoms. Respondents who were married or had a higher household income had lower levels of depressive symptoms. 

In terms of self-rated health, older adults in the restricted, family-restricted or friends network displayed significantly lower levels of self-rated health than those in the diverse network (*p* < 0.05 or *p* < 0.001). However, there was no significant difference between the family network and diverse network in the levels of self-rated health. In addition, the level of self-rated health of men were higher than those of women, and older adults with a higher income had higher levels of self-rated health.

### 3.3. Analysis of the Mediating Effect of Social Support

To examine whether social support played a mediating role in the relationship between the social network types and health, we first conducted multiple linear regression analysis with social support as the dependent variable and social network types as the independent variables to see whether the social network types were significantly associated with social support. As shown in Table 5, the beta coefficients relating social network types to social support were significant (*p* < 0.001). Older adults in the diverse network had the most social support compared with those in other network types, but the restricted network had the least. In addition, older people who were married and had higher incomes received more social support.

The confirmation that the social network types were significantly associated with social support brought social support further into the regression equation of social network types and health. The significant beta coefficients relating the social network types to depressive symptoms and self-rated health were reduced after we included social support in the model (Table 6). As mentioned before, older adults in the diverse network did not differ significantly in depressive symptoms from those in the friends network, nor did they differ in self-rated health from those in the family network; however, for the other three network types, differences in social support appear to at least partially explain the differences from the diverse network in depressive symptoms and self-rated health. In addition, social support was significantly associated with depressive symptoms and self-rated health (*β* = −0.60, *p* < 0.001; *β* = 0.28, *p* < 0.001). This confirmed that social support played a partial mediating role in the association of social network types with depressive symptoms and self-rated health: the more social support older adults received, the better their condition with respect to depressive symptoms and self-rated health.

## 4. Discussion

### 4.1. Social Network Clustering Analysis

This study identified five robust network types: diverse, restricted, family, friends, and a specific type—family-restricted. The formation of the family-restricted network is related to the unique social environment in rural China. The social network size of the family-restricted network is smaller than that of the family network, and older adults in this network rarely have contact with members of their social network, except for contact with living children. Besides, individuals with this network are older than other network types. Generally, as age increases, older adults are more limited in energy and incline to shrink their social networks, and only maintain the most intimate kinship ties [41,42]. The family network accounts for the largest proportion in this study due to the fact that most of rural older adults have an above average number of children and mainly contact their children, but have relatively little contact with other social members and social activities. The reason may be that males accounted for the largest proportion in the this network, and they are the main labor force in rural areas; therefore, they are usually busy doing farm work, except for New Year’s Day, other holidays, or weddings. 

In the friends network, the number of close friends and the frequency of contact with them are the highest, and the value of the frequency of social activities is relatively high. However, the value of the number of living children is lower than other network types, except for the restricted network. The individuals in this network are relatively young and have more energy. Additionally, they may have a pleasant personality and like to participate in social activities. Diverse and restricted are two networks with opposite characteristics. The former has a large social network and frequent social exchanges; however, the latter has a very small social network and basically does not interact. The proportion of the restricted network is less than that of the family-restricted network, mainly because rural older adults generally have contact with their children, except for some single ones without children.

Although the diverse, restricted, family and friends network types we identify have been consistently found in other countries, there are still differences in the details among these common types. In this study, the proportion of the family network greatly exceeds that of the restricted network, but the proportion of the family network is still smaller than that of the restricted network in the United States [5,10] and South Korea [19]. In this study, the restricted network is characterized by low values for all social network variables, which is not consistent with other studies in Western countries, such as the United States [5,12] and Australia [14]; for example, the number of friends was higher in the restricted network than the family network. Besides, individuals with a friends network have fewer interactions with family members than other network types [14], which is different from the findings in our study.

### 4.2. Analysis of the Association between Social Network Type and Health

First, the social network types are significantly associated with depressive symptoms, older people with diverse, and friends networks were significantly associated with lower levels of depressive symptoms, while the opposite was true for those older adults in restricted and family-restricted networks. This result is consistent with previous studies, which indicated a diverse network is more favorable in mental health than a restricted network [10,16,17,18,19]. The depressive symptoms of older adults in the family network are more serious than that of those in the friends network, which is inconsistent with a study in Germany [13]. The reason may be that the frequency of contact with friends and participation for individuals in the friends network are significantly higher than for those in the family network, and more social interaction is an important factor in relieving depression [5,18]. This also explains why depressive symptoms for individuals in the family-restricted network are more severe than for those in the family network. Some scholars have indicated that spontaneous or voluntary relationships among friends are more conducive to mental health than the responsibility relationships that bind family members [43]. This further illustrates that with economic development and its attendant social structure changes, rural older adults are no longer confined to the family environment and are constantly improving their social network relationships. For example, older adults more frequently interact with friends and participate in more social activities with their friends. 

Second, the social network types are also significantly associated with self-rated health. Older adults with restricted and family-restricted networks were associated with lower levels of self-rated health than those with other networks. This conclusion is consistent with previous studies [16,18]. In this study, the family network is found to be slightly better than the friends network for self-rated health when compared with the diverse network. This result is inconsistent with the studies conducted in the United States [12] and South Korea [18]. The reason for this finding may be that friends generally offer more information and emotional support, which are more beneficial to mental health. Family members provide more tangible support, which is of benefit to physical health [27,33,44]. 

### 4.3. Analysis of the Mediating Effect of Social Support

Social support plays a partial mediating role in the relationship between the social network types and health. This result is not consistent with the study of Webster et al. [26], which found that social support did not mediate the link between social network types and depressive symptoms. The possible reasons are that individuals have differences for their social relationships in diverse cultural contexts. Additionally, differences of the adopted social network variables and social support scale may also have some impacts on the results. The social support scale used in this study mainly reflects the frequency of social support. Social network types are closely related to social support. A diverse network has more network members and more frequent contact, so older adults in this type tend to receive more social support. In restricted networks, on the contrary, social network members are extremely few, so the social support is less [28,33]. The social network size and participation in social activities offered by the family network are lower than those of the friends network, so the social support obtained is relatively little for older adults in the family network. Furthermore, social support is significantly associated with health. The more social support available, the more likely an older adult has lower level of depressive symptoms, and the better his or her self-rated health will be. Many studies have analyzed the relationship between social support and health, the greater the social support, the more difficult it is for older people to feel lonely, and loneliness is closely related to depression [45,46]. Social support can also promote health by inhibiting physiological stress responses [47].

### 4.4. Limitations and Future Directions

First of all, this study uses cross-sectional survey data. In the future, longitudinal data or queue tracking research could be used to analyze the causal relationship between social network type and health, thus capturing the characteristics and changes of social network characteristics of older people at different stages. Secondly, based on the lack of research into the relationship between social network type and health of rural older adults, the survey sample of this paper included only rural older adults. The urban older adults should be included in the future, and we would recommend comparing the differences between the network types of the urban and rural older adults. In addition, social support entails multiple dimensions, and the next step can be to analyze the impacts of social network types on different dimensions of social support. At the same time, some mediator variables could be added, such as perceived quality of social relations, and a powerful statistical technique—structural equation modeling would be adopted to further clarify the mechanism of the impact of social network types on health.

## 5. Conclusions

This study used a cross-sectional survey approach to obtain data on the social network relationships of rural older adults and successfully identified five robust social network types: diverse, restricted, family, friends, and a specific type—family-restricted—that has not been previously described. 

Furthermore, these social network types were significantly associated with depressive symptoms and self-rated health. In addition, our study indicated that social support played a partial mediating role in the relationship between social network types and health. This study provides empirical reference for scholars from various countries in the field of the relationship between social network types and health. With respect to the association mechanism between social network types and health, the present study may scientifically guide decision-makers in taking effective measures to improve the social network relationships of older adults and promote healthy aging.

## Figures and Tables

**Table 1:** Distribution of social network types and characteristics of criterion variables (*N* = 405).

Criterion Variables	Social Network Types				
	Diverse (84/20.74%)	Restricted (54/13.33%)	Family-Restricted (66/16.30%)	Family (111/27.41%)	Friends (90/22.22%)
Number of living children	3.75(0.81)	*1.48(0.69)*	**4.08(0.85)**	3.29(1.21)	2.07(0.47)
Number of close relatives	**3.64(0.53)**	*2.04(0.55)*	2.65(0.57)	3.21(0.61)	3.40(0.60)
Number of close friends	2.65(0.70)	*0.39(0.56)*	0.47(0.50)	1.80(0.64)	**2.68(0.76)**
Frequency of contact: living children	**4.02(0.41)**	*2.33(0.99)*	3.48(0.71)	3.96(0.69)	3.58(0.67)
Frequency of contact: close relatives	**2.88(0.61)**	*1.57(0.50)*	1.85(0.64)	2.24(0.62)	2.42(0.65)
Frequency of contact: close friends	3.49(0.69)	*0.46(0.69)*	0.65(0.81)	2.81(0.55)	**3.94(0.71)**
Frequency of social activities	**3.85(0.78)**	*1.07(0.26)*	1.11(0.36)	1.39(0.58)	3.41(0.78)

Notes: Means with standard deviations in parentheses. The highest mean value is presented in bold, and the lowest mean value is italicized. Criterion variable scale ranges: Number of living children/close relatives/close friends (0–5); Frequency of contact with living children/close relatives/close friends (1–5); Frequency of social activities (1–5).

**Table 2:** Sociodemographics, depressive symptoms, and self-rated health by social network type.

Characteristic	Social Network Types					*χ*^2^/*F*	*p*	Post Hoc Tests of Means ^*a*^
	Diverse	Restricted	Family-Restricted	Family	Friends			
Age (%)								
60–69	70.24	44.44	24.24	55.86	78.89	χ^2^ = 55.97		
≥70	29.76	55.56	75.76	44.14	21.11		<0.001	
Gender (%)								
Male	40.48	51.85	31.82	54.05	46.67	χ^2^ = 10.00		
Female	59.52	48.15	68.18	52.34	53.33		0.040	
Marital status (%)								
Married	91.67	48.15	71.21	82.88	91.11	χ^2^ = 51.97		
Not married	8.33	51.85	28.79	17.12	8.89		<0.001	
Educational level (%)								
No education	27.38	51.85	57.58	46.85	32.22	χ^2^ = 23.11		
Primary school	48.81	40.74	34.85	40.54	54.44			
Middle school or beyond	23.81	7.41	7.58	12.61	13.33		<0.001	
Household income (%)								
<5000	20.24	61.11	59.09	32.22	14.44	χ^2^ = 72.27		
5000–15,000	19.05	25.93	27.27	40.54	36.67			
15,000–25,000	26.19	7.41	7.58	19.82	30.00			
≥25,000	34.52	5.56	6.06	13.51	18.89		<0.001	
Depressive symptoms, M (SD)	2.88(2.80)	10.59(3.06)	8.92(3.43)	5.36(3.94)	3.66(2.76)	*F* = 70.60	<0.001	1, 5 < 2, 3, 4 2, 3 > 1, 4, 5
Self-rated health, M (SD)	3.29(0.72)	2.26(0.68)	2.30(0.84)	3.06(0.87)	3.01(0.77)	*F* = 24.82	<0.001	2, 3 < 1, 4, 5

Notes: *^a^* 1 = diverse network, 2 = restricted network, 3 = family-restricted network, 4 = family network, 5 = friends network.

**Table 3:** Depressive symptoms and self-rated health by sociodemographics: Spearman correlations.

Variables	Depressive Symptoms		Self-Rated Health	
	*r*	*p*	*r*	*p*
Age *^a^*	0.38	<0.001	−0.27	<0.001
Gender *^b^*	0.11	0.028	−0.18	<0.001
Marital status *^c^*	0.40	<0.001	−0.25	<0.001
Educational level *^d^*	−0.32	<0.001	0.25	<0.001
Household income *^e^*	–0.55	<0.001	0.40	<0.001

Notes: *^a^* 1 = 60–69, 2 = Age ≥ 70; *^b^* 1 = male, 2 = female; *^c^* 1 = married, 2 = not married; *^d^* 1 = no education, 2 = primary school, and 3 = middle school or beyond; *^e^* 1 = less than 5000, 2 = 5000–15,000, 3 = 15,000–25,000, and 4 = not less than 25,000.

**Table 4:** Influence of social network types on depressive symptoms and self-rated health.

Variables	Depressive Symptoms			Self-Rated Health		
	b (*SE*)	*β*	*p*	b (*SE*)	*β*	*p*
Social network types						
Diverse	-	-		-	-	
Restricted	5.86(0.56)	0.47	<0.001	−0.82(0.14)	−0.32	<0.001
Family-restricted	4.28(0.52)	0.37	<0.001	−0.69(0.13)	−0.29	<0.001
Family	1.74(0.44)	0.18	<0.001	−0.13(0.11)	−0.07	0.243
Friends	0.53(0.46)	0.05	0.246	−0.25(0.12)	−0.12	0.030
Sociodemographics						
Age	0.18(0.37)	0.02	0.624	−0.11(0.09)	−0.06	0.251
Gender	0.61(0.34)	0.07	0.069	−0.24(0.09)	−0.14	0.005
Marital status	1.02(0.43)	0.10	0.018	0.06(0.11)	0.03	0.595
Educational level	−0.39(0.26)	−0.06	0.132	−0.05(0.07)	−0.04	0.481
Household income	−1.08(0.18)	−0.27	<0.001	0.18(0.05)	0.22	<0.001
Adjusted *R*-square	0.52		<0.001	0.26		<0.001

Note: SE: standard error.

**Table 5:** Influence of social network types on social support.

Variables	Social Support		
	b (*SE*)	*β*	*p*
Social network types			
Diverse	-	-	
Restricted	−0.88(0.06)	−0.63	<0.001
Family-restricted	−0.52(0.05)	−0.40	<0.001
Family	−0.35(0.05)	−0.33	<0.001
Friends	−0.19(0.05)	−0.17	<0.001
Sociodemographics			
Age	0.05(0.04)	0.05	0.182
Gender	−0.01(0.03)	−0.01	0.686
Marital status	−0.24(0.04)	−0.20	<0.001
Educational level	0.05(0.03)	0.07	0.086
Household income	0.10(0.02)	0.21	<0.001
Adjusted *R*-square	0.60		<0.001

**Table 6:** Influence of social network types and social support on depressive symptoms and self-rated health.

Variables	Depressive Symptoms			Self-Rated Health		
	b (*SE*)	*β*	*p*	b (*SE*)	*β*	*p*
Step 1						
Diverse	-	-		-	-	
Restricted	5.86(0.56)	0.47	<0.001	−0.82(0.14)	−0.32	<0.001
Family-restricted	4.28(0.52)	0.37	<0.001	−0.69(0.13)	−0.29	<0.001
Family	1.74(0.44)	0.18	<0.001	−0.13(0.11)	−0.07	0.243
Friends	0.53(0.46)	0.05	0.246	−0.25(0.12)	−0.12	0.030
Step 2						
Diverse	-	-		-	-	
Restricted	1.16(0.60)	0.10	0.052	−0.38(0.18)	−0.15	0.035
Family-restricted	1.52(0.49)	0.13	0.002	−0.43(0.15)	−0.18	0.003
Family	−0.12(0.40)	−0.01	0.763	0.04(0.12)	0.02	0.714
Friends	−0.50(0.39)	−0.05	0.199	−0.16(0.12)	−0.07	0.182
Social support	−5.36(0.42)	−0.60	<0.001	0.51(0.12)	0.28	<0.001
△*R*^2^	0.14		<0.001	0.03		<0.001
